# Assessment of predation risk through conspecific cues by anuran larvae

**DOI:** 10.1007/s10071-023-01793-y

**Published:** 2023-06-07

**Authors:** Carlos Caballero-Díaz, Rosa Arribas, Nuria Polo-Cavia

**Affiliations:** 1 Department of Biology, Universidad Autónoma de Madrid, Ciudad Universitaria de Cantoblanco, 28049 Madrid, Spain; 2 Present Address: Monitoring Team on Natural Processes ICTS-RBD, Estación Biológica de Doñana (CSIC), Calle Américo Vespucio 26 - Isla de la Cartuja, 41092 Sevilla, Spain

**Keywords:** Antipredator behavior, Disturbance cues, Larval amphibians, *Pelobates cultripes*, Risk assessment, Social learning

## Abstract

Accurate assessment of predation risk is critical for prey survival during predator–prey interactions. Prey can assess predation risk by the presence of cues dropped by predators themselves, but they can also gather information about risk level through cues released by other prey, avoiding the hazard of being in close proximity to predators. In this study, we examine the ability of anuran larvae (*Pelobates cultripes*) to detect predation risk indirectly when they are in contact with conspecifics that have been recently exposed to chemical stimuli from natural predators (larvae of aquatic beetles). In a first experiment, we confirmed that larvae exposed to predator cues exhibited innate defensive behavior, indicating that they perceived the risk of predation and, thus, could potentially act as risk indicators for naïve conspecifics. In a second experiment, we observed that unexposed larvae paired with a startled conspecific adjusted their antipredator behavior, presumably by mirroring conspecifics’ behavior and/or using chemical cues from their partners as a risk information source. This cognitive ability of tadpoles to assess predation risk through conspecific cues might play an important role in their interaction with predators, facilitating the early detection of potential threats to elicit appropriate antipredator responses and increase the chances of survival.

## Introduction

Predation pressure is one of the major selective forces in nature (Vermeij 1982). Consequently, prey species have evolved a wide variety of defensive strategies to evade predators, which can involve changes in morphology, physiology, behavior, and/or life history (Benard 2004; Lima and Dill 1990; Tollrian and Harvell 1999). Because antipredator responses generally entail important energetic and/or opportunity costs (Chivers and Smith 1998; Chivers et al. 2001; Helfman 1989), defenses are often inducible, expressed only in the presence of predators (Clark and Harvell 1992; Tollrian and Harvell 1999). Activation of plastic defenses thus hinges on risk perception, requiring prey to accurately assess predation threats and flexibly employ antipredator tactics according to changing background risk (Brown et al. 2006; Martín and López 2005; Sih 1997).

By collecting the first-hand information dropped by predators, prey can dynamically assess predation risk, but it might be very dangerous (Chivers and Smith 1998; Kats and Dill 1998; Stauffer and Semlitsch 1993). Alternatively, prey can gather information about the risk level indirectly, through social cues released by other prey (Ferrari et al. 2010; Schoeppner and Relyea 2009). For instance, less-experienced individuals can learn novel information and acquire or enhance antipredator responses by observing more experienced individuals, or by associating certain stimuli with predatory attacks toward other prey. Such social learning plays an important role in the early detection of predation risk, allowing threat-sensitive adjustments of behavioral decisions without incurring the costs of direct exposure to potential predators, hence being crucial for prey survival (Helfman 1989; Manassa and McCormick 2012; Mirza and Chivers 2002). Social learning in the context of predation has been observed in a wide range of taxa (reviewed in Griffin 2004; Crane and Ferrari 2013; Griffin 2004), although the vast majority of studies have focused on mammals, birds, and schooling fishes. In contrast, little information exists regarding other vertebrates such as amphibians (but see Chivers and Ferrari 2014; Ferrari and Chivers 2008; Ferrari et al. 2007).

Amphibians are especially sensitive to predation during the early stages of their development, when predators consume many embryos and larvae (Duellman and Trueb 1994; Wells 2010). Larvae of many amphibian species typically respond to the presence of predators by developing defensive phenotypes (Gomez-Mestre and Diaz-Paniagua 2011; Relyea 2004; Smith and Van Buskirk 1995; Van Buskirk 2009), or by reducing their activity (Holomuzki 1995; Kats and Dill 1998; Kiesecker et al. 1996; Polo-Cavia and Gomez-Mestre 2014; Polo-Cavia et al. 2010;). Though amphibian larvae do not display collective defensive behavior, they are known to often form dense aggregations and swim together in the same direction (Beiswenger 1975; Blaustein and O’Hara 1986). Thus, using social cues (i.e., behavior and/or chemical cues) informing about predation risk and mirroring other individuals, aggregated tadpoles might dynamically adjust their antipredator responses to background level of risk and benefit from social transmission of fine-tuned defensive behaviors among shoal companions (Ferrari et al. 2007; Hall and Suboski 1995; Hoppitt and Laland 2013; Suboski et al. 1990; Wilson et al. 2021).

The use of social cues in cultural transmission of predator recognition was first demonstrated in larval amphibians by Ferrari et al. (2007), by pairing naïve tadpoles of *Lithobates sylvatica* with experienced conspecifics responding to the scents of predatory tiger salamanders (*Ambystoma tigrinum*). Shortly after, Ferrari and Chivers (2008) proved that naïve tadpoles of another frog, *Pseudacris maculate*, were also capable of labeling this predator by observing the antipredator responses of *L. sylvatica* tadpoles. In the last decades, a growing number of studies have suggested that disturbance cues (i.e., chemicals released by uninjured prey when they detect a predator or feel stressed; Wisenden 2015) might function as information sources for risk assessment in aquatic habitats, potentially triggering predator avoidance responses in nearby individuals (Chivers and Smith 1998; Ferrari et al. 2008; Goldman et al. 2020; Wisenden et al. 1995; Wisenden 2000; see Crane et al. 2022 for an extended review). However, these studies are also scant in the amphibian literature, with a few anuran species responding to disturbance cues (Bairos-Novak et al. 2017, 2019; Gonzalo et al. 2010; Kiesecker et al. 1999; Manteifel et al. 2005; Rivera-Hernández et al. 2022). Here, we examine the capacity of naïve larvae of the western spadefoot toad, *Pelobates cultripes*, to assess predation risk through social cues from conspecifics that were previously in contact with scents from natural predators (larvae of aquatic Dytiscidae). We hypothesize that visual and/or chemical cues (i.e., antipredator behavior and/or disturbance cues) released by larvae recently exposed to the predator scents might act as information sources of predation risk for naïve conspecifics. Hence, we expected larvae unexposed to predator cues to adjust their antipredator behavior influenced by experienced larvae previously exposed to these cues.

## Materials and methods

### Study animals

We collected three egg clutches of *Pelobates cultripes* by sampling different ponds and streams in Colmenar Viejo (Madrid province, central Spain). Eggs (< 10 Gosner; Gosner 1960) were transported to Doñana Biological Station in Seville and housed in a walk-in climatic chamber to guarantee naïvety of experimental tadpoles to predator cues. Upon hatching, larvae were raised individually in 3 L plastic buckets with carbon-filtered dechlorinate tap water at 20 ºC and under a natural photoperiod (12:12 light:darkness). We renewed the water every 2 days, and subsequently, we fed larvae with ground rabbit chow and lightly boiled spinach.

We also collected larvae of aquatic beetles (Dytiscidae), which are common predators of amphibian larvae (Brodie and Formanowicz 1983), to be used as predator cue donors in the experiments. Donor beetle larvae were housed individually in 1 L plastic buckets in a climatic chamber separated from that of amphibians to avoid any chemical or visual contact prior to the experiments. Beetle larvae were fed one or two *P. cultripes* tadpoles from a stock tank every other day, and temperature and photoperiod conditions were the same as those of amphibians. All surviving tadpoles were kept until metamorphosis and released as juveniles at their ponds of origin after standard prophylaxis procedures, whereas no beetle larvae survived to the experiments.

### Preparation of chemical stimuli

To prepare predator chemical cues, we filled each donor beetle bucket with 0.5 L of dechlorinated tap water. Since predator cues last approximately 2–4 days in water (Peacor 2006), we waited 2 days and then blended the water from five donor buckets pervaded with predator cues, filtered it, and immediately froze it in 10 mL aliquots to be used in the experiments. We also prepared 10 mL aliquots of clean water following the same procedure but without placing predators in the buckets (Polo-Cavia and Gomez-Mestre 2014; Polo-Cavia et al. 2010).

## Experimental procedure

We examined the capacity of amphibian larvae to assess predation risk through conspecific cues by performing an experiment in which we compared larval behavior under different risk conditions. Prior to the experiment, larvae were marked with Visible Implant Elastomer (VIE) tags (Northwest Marine Technology, Inc.), so we could identify them during the trials. VIE tags were injected subcutaneously in the dorsal area of larval body using a 29-G insulin syringe (BD Micro-Fine Insulin U-100 0.5 mL) (Burraco et al. 2017). We used a single color (red) and randomly marked half of the larvae in each treatment to avoid introducing potential biases in larval responses among treatments. After the VIE tag injection, marked larvae were monitored during the following 24 h in 10 L aquaria; 100% of larvae survived.

**Fig. 1:**
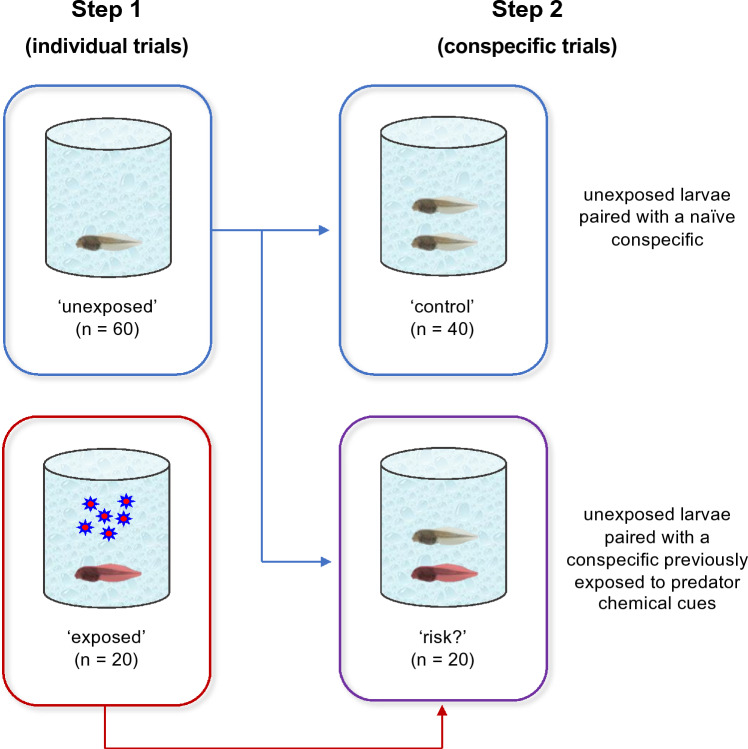
Experimental design of the study. In a first step (*Step 1*), larvae were individually exposed to clean water (‘unexposed’ treatment) or to water with chemical cues from predators (‘exposed’ treatment). In a second step (*Step 2*), unexposed larvae from *Step 1* were paired with a naïve conspecific (‘control’ treatment) or with a conspecific previously exposed to chemical cues (‘risk?’ treatment) and all pairs (*n* = 40) were tested in clean water. With this experimental design, we assessed (1) whether larvae in the ‘exposed’ treatment responded to chemical cues from predators and therefore could be used as indicators of predation risk for conspecifics, and (2) whether larvae in the ‘risk?’ treatment were able to perceive predation risk without being exposed to predator cues, but induced by the presence of a conspecific from the ‘exposed’ treatment which had been recently exposed to such cues

In a first step (*Step 1*), 60 larvae were randomly assigned to the ‘unexposed’ treatment and 20 were assigned to the ‘exposed’ treatment. Larvae in the ‘unexposed’ treatment were individually exposed to clean water, whereas larvae in the ‘exposed’ treatment were individually exposed to water with chemical cues from predators (Fig. 1). In a second step (*Step 2*), we split larvae from the ‘unexposed’ treatment into two groups: 40 larvae were randomly assigned to the ‘control’ treatment and 20 were assigned to the ‘risk?’ treatment. Each larva from the ‘control’ treatment was paired with another larva from the same treatment, whereas each larva from the ‘risk?’ treatment was paired with a larva from the ‘exposed’ treatment, and all pairs (n = 40) were exposed to clean water (Fig. 1). Thus, naïve larvae in the ‘control’ and ‘risk?’ treatments were sequentially observed in two testing treatments, i.e., individually in *Step 1* and with a conspecific in *Step 2* (‘individual’ vs. ‘conspecific’), whereas larvae in the ‘exposed’ treatment were observed only individually in *Step 1*. With this experimental design, we assessed 1) whether larvae in the ‘exposed’ treatment responded to chemical cues from predators and therefore could be used as indicators of predation risk for conspecifics, and 2) whether larvae in the ‘risk?’ treatment were able to perceive predation risk without being exposed to predator cues, but induced by the presence of a conspecific from the ‘exposed’ treatment which had been recently exposed to such cues. The observation of antipredator behavior by tadpoles in the presence of predator cues or a startled conspecific, in comparison with the baseline behavior of tadpoles tested in clean water or in the presence of a naïve conspecific, was considered as indicative of risk assessment. Also, by including the ‘control’ treatment, we could disentangle the effect of the presence of a conspecific per se on larval behavior from its role in social transmission of risk information among tadpoles.

Trials were conducted in 1 L plastic buckets with dechlorinated tap water. Depending on the treatment, we added to each bucket 10 mL aliquot of clean water (‘unexposed’ treatment) or 10 mL aliquot of water with the predator cues (‘exposed’ treatment). Since beetle larvae were fed conspecific tadpoles, larvae in the ‘exposed’ treatment received a mix of predator odor (kairomone) joint with post‐digestion alarm cues from consumed conspecifics, which was expected to increase the level of risk perceived and to reinforce the antipredator responses of larvae. Larvae were recorded using a video camera (Sony HDR-SR11E) for 30 min in the ‘individual’ treatment (*Step 1*), and then, pairs were immediately transferred to a different bucket and recorded for another 30 min in the ‘conspecific’ treatment (*Step 2*). Before being transferred to the conspecific trials, larvae from both unexposed and exposed conditions were rinsed in clean water to ensure that no residual chemical cues from predators or tadpole alarm cues influenced the antipredator responses of naïve larvae. The ‘individual’ treatment was always applied first to avoid biasing larval baseline behavior.

Larvae were given a brief time for acclimation to the experimental buckets at the beginning of the trials, so we discarded the first 5 min of each video. We then blindly monitored each larva for 25 min, using the instantaneous scan sampling method, recording the following behavioral variables every 30 s: (1) swimming activity (active vs. motionless), (2) caudal vibration, i.e., tail movement without larval displacement (moving vs. not moving), (3) wall scratching behavior (scratching vs. not scratching), and (4) the position of the larva in the water column (top vs. bottom). We also counted (5) the number of times each larva took air from the surface and (6) the number of darting movements by each larva. These variables can be used as indicators of perceived predation risk among larval amphibians, since they are easily recognizable fear-related behaviors that may act as social cues, and that have been observed to become altered in a predation context (Griffin 2004; Holomuzki 1995; Kats and Dill 1998; Kiesecker et al. 1996; Peters et al. 2002; Stauffer and Semlitsch 1993; Wilson and Lefcort 1993).

### Data analyses

To analyze whether larvae exposed to chemical cues from predators were able to recognize the risk of predation, we compared the behavior of unexposed and exposed larvae in individual trials (*Step 1*) by conducting a multivariate analysis of variance (MANOVA) with the behavioral responses of larvae (swimming activity, caudal vibration, wall scratching, position in the water column, surfacing, and darting) as dependent variables and exposure to predator cues (‘unexposed’ vs. ‘exposed’) as a between-subject factor. To analyze whether naïve larvae not exposed to chemical cues from predators were able to assess predation risk through conspecifics, we compared the behavior of larvae in the ‘control’ and ‘risk?’ treatments across individual (*Step 1*) and conspecific (*Step 2*) trials by performing two-way repeated-measures analyses of variance (ANOVAs) with each behavioral variable as the dependent variable, risk treatment (‘control’ vs. ‘risk?’) as a between-subject factor, and testing treatment (‘individual’ vs. ‘conspecific’) as a within-subject factor.

We verified data normality and homogeneity of variances using Kolmogorov–Smirnov and Levene’s test, respectively. Mean comparisons among treatments were performed using protected Fisher’s LSD tests (Sokal and Rohlf 1995). Statistical analyses were performed with Statistica 12.0 (Statsoft, Tulsa, OK).

## Results

### Responses to predator chemical cues

**Fig. 2:**
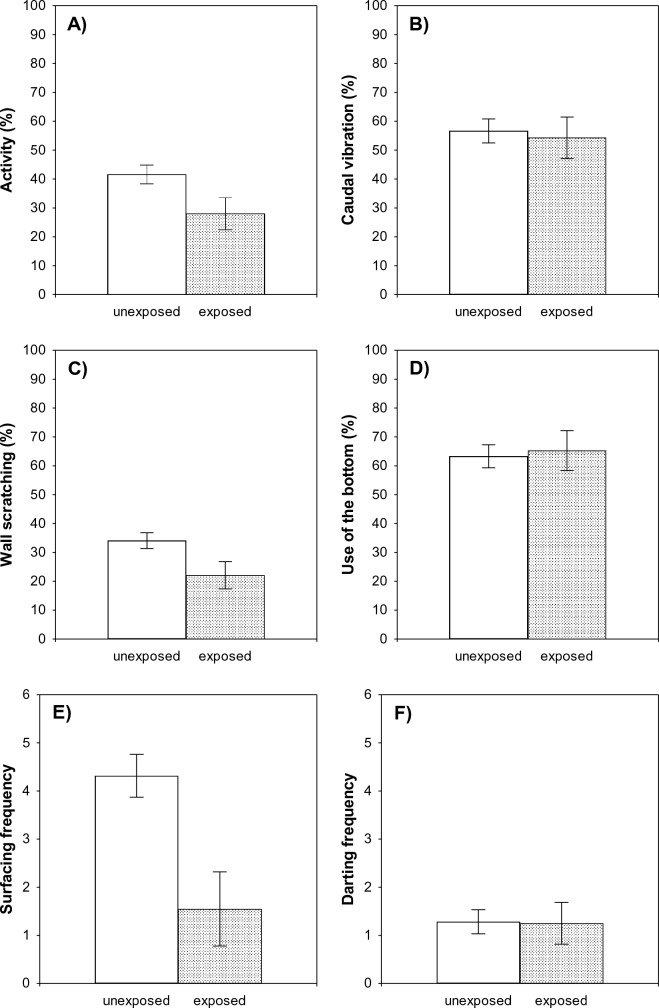
Behavioral responses of unexposed () and exposed () larvae to predator chemical cues in individual trials (*Step 1*). In this step of the experiment, we assessed whether larvae exposed to chemical cues from predators were able to recognize the threat and, therefore, could be used as indicators of predation risk for conspecifics in paired trials in a second step (*Step 2*) of the experiment. The observation of antipredator behavior by tadpoles in the presence of predator cues was considered as indicative of risk assessment. **A** Swimming activity, **B** caudal vibration, **C** wall scratching, **D** use of the bottom, **E** surfacing, and **F** darting. Bars represent mean ± SE for each group

The multivariate analyses showed a significant effect of exposure to chemical cues from predators on larval behavior (MANOVA, *F*_6,73_ = 3.68, *p* = 0.003), thus indicating that larvae in the ‘exposed’ treatment did perceive the risk of predation. Larvae exposed to predator cues significantly reduced their swimming activity compared to that of unexposed larvae in clean water (ANOVA, *F*_1,78_ = 4.43, *p* = 0.04, Fig. 2A). Caudal vibration did not significantly differ between larvae exposed and not exposed to predator cues (*F*_1,78_ = 0.08, *p* = 0.78, Fig. 2B), but larvae exposed to predator cues spent significantly less time scratching the walls of the experimental bucket than unexposed larvae (*F*_1,78_ = 4.82, *p* = 0.03, Fig. 2C). Position in the water column did not significantly vary between exposed and unexposed larvae (*F*_1,78_ = 0.06, *p* = 0.80, Fig. 2D). Frequency of surfacing was significantly higher in unexposed than in exposed larvae (*F*_1,78_ = 9.67, *p* = 0.003, Fig. 2E), but the effect of predator cues on frequency of darting was not significant (*F*_1,78_ = 0.004, *p* = 0.95, Fig. 2F).

### Assessment of predation risk through conspecifics

#### Swimming activity

**Fig. 3:**
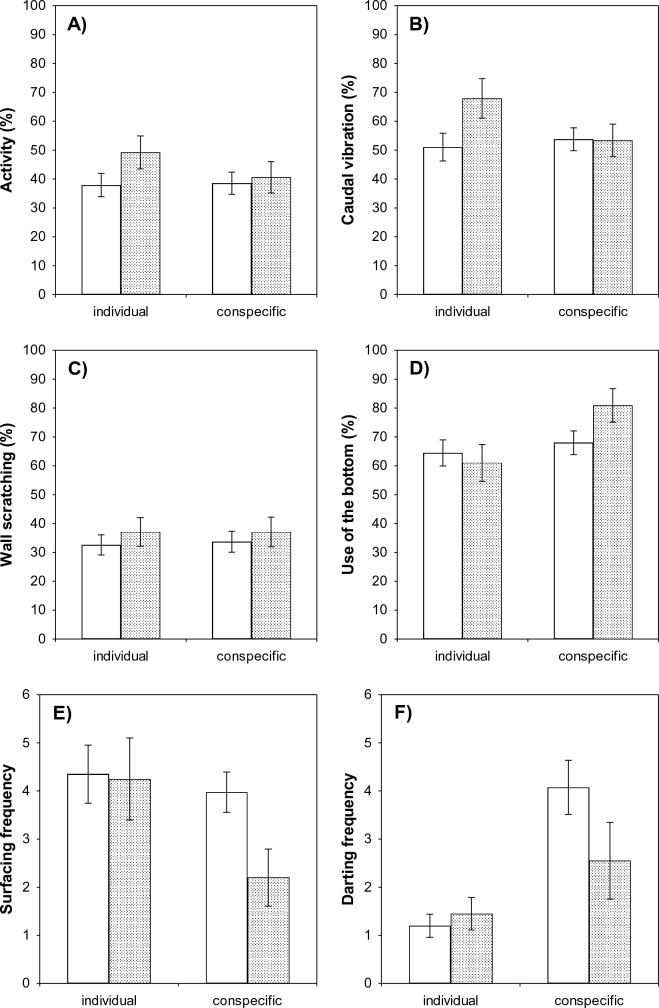
Behavioral responses of unexposed larvae, ‘control’ () and ‘risk?’ () treatments, in individual trials (*Step 1*) and in trials with a conspecific (*Step 2*). For the conspecific trials, larvae in the ‘control’ treatment were paired with a naïve conspecific, whereas larvae in in the ‘risk?’ treatment were paired with a conspecific previously exposed to predator chemical cues. By comparing behavior of larvae in these two treatments across individual and conspecific trials, we assessed whether naïve tadpoles not exposed to predator cues were able to assess predation risk induced by the presence of a startled conspecific recently exposed to such cues. Notice that the effect of conspecifics per se on larval behavior does not indicate social transmission of risk; the observation of antipredator behavior in the presence of a startled conspecific, in comparison with a naïve one, does indicate risk information transfer instead. **A** Swimming activity, **B** caudal vibration, **C** wall scratching, **D** use of the bottom, **E** surfacing, and **F** darting. Bars represent mean ± SE for each group

Overall larval swimming activity was not significantly affected by risk treatment (‘control’ vs. ‘risk?’) or by the presence of a conspecific during the trials (two-way repeated-measures ANOVA, *F*_1,58_ = 1.13, *p* = 0.29 and *F*_1,58_ = 2.25, *p* = 0.14, respectively). The effect of the interaction between risk treatment and conspecific presence showed a trend (*F*_1,58_ = 3.04, *p* = 0.09) (Fig. 3A). The presence of a conspecific did not affect activity of larvae in the ‘control’ treatment, whereas larvae in the ‘risk?’ treatment tended to reduce their activity in the presence of a conspecific, compared to their activity in individual trials. Activity was similar between treatments, either within individual trials or within trials with a conspecific.

#### Caudal vibration

Overall tail movement of larvae was not significantly affected by risk treatment (two-way repeated-measures ANOVA, *F*_1,58_ = 1.41, *p* = 0.24), but the presence of a conspecific during the trials had a nearly significant effect on overall tail movement (*F*_1,58_ = 3.39, *p* = 0.07), which tended to be higher in individual trials (mean ± SE = 59.5 ± 4.2%) than in trials with a conspecific (53.6 ± 3.4%). The effect of the interaction between risk treatment and conspecific presence was significant (*F*_1,58_ = 7.21, *p* = 0.009) (Fig. 3B). The presence of a conspecific did not significantly affect tail movement of larvae in the ‘control’ treatment (Fisher’s LSD, *p* = 0.47), whereas larvae in the ‘risk?’ treatment significantly reduced caudal vibration in the presence of a conspecific (*p* = 0.008). When larvae were tested individually, caudal vibration was significantly higher in the ‘risk?’ treatment than in the ‘control’ treatment (*p* = 0.03), but there were no significant differences between treatments within trials with a conspecific (*p* = 0.96).

#### Wall scratching

Overall time that larvae spent scratching the walls of the experimental bucket was not significantly affected by risk treatment (two-way repeated-measures ANOVA, *F*_1,58_ = 0.48, *p* = 0.49) or the presence of a conspecific (*F*_1,58_ = 0.06, *p* = 0.82), and the interaction between these two factors also had no significant effect on wall scratching (*F*_1,58_ = 0.06, *p* = 0.82) (Fig. 3C).

#### Position in the water column

Risk treatment did not significantly affect the position of larvae in the water column overall (two-way repeated-measures ANOVA, *F*_1,58_ = 0.58, *p* = 0.45), but the effects of the presence of a conspecific and its interaction with risk treatment were significant (*F*_1,58_ = 7.98, *p* = 0.007) and on the edge of significance (*F*_1,58_ = 3.88, *p* = 0.05), respectively (Fig. 3D). Larvae occupied preferably the bottom part of the water column (mean ± SE = 68.6 ± 2.5%) and spent more time in the bottom in trials with a conspecific (74.5 ± 3.6%) than in individual trials (62.7 ± 3.9%) overall. The presence of a conspecific did not significantly affect the position in the water column of larvae in the ‘control’ treatment (Fisher’s LSD, *p* = 0.46), whereas larvae in the ‘risk?’ treatment spent more time in the bottom part of the experimental bucket in the presence of a conspecific than in individual trials (*p* = 0.005). Use of the bottom was similar between treatments when larvae were tested individually (*p* = 0.65), but within trials with a conspecific, larvae in the ‘risk?’ treatment tended to spend more time in the bottom than larvae in the ‘control’ treatment (although differences did not reach significance, *p* = 0.09).

#### Surfacing

The effect of risk treatment on overall surfacing behavior of larvae was not significant (two-way repeated-measures ANOVA, *F*_1,58_ = 2.27, *p* = 0.14), but the presence of a conspecific had a nearly significant effect on surfacing frequency (*F*_1,58_ = 3.49, *p* = 0.07), which tended to be higher in individual trials (mean ± SE = 4.3 ± 0.5) than in trials with a conspecific (3.1 ± 0.4) overall (Fig. 3E). The effect of the interaction between conspecific presence and risk treatment was not significant (*F*_1,58_ = 1.67, *p* = 0.20).

#### Darting

Overall frequency of darting movements by larvae was not affected by risk treatment (two-way repeated-measures ANOVA, *F*_1,58_ = 1.10, *p* = 0.30), but the presence of a conspecific and its interaction with risk treatment had significant effects on darting behavior (*F*_1,58_ = 20.78, *p* < 0.0001 and *F*_1,58_ = 4.14, *p* = 0.04 respectively) (Fig. 3F). Overall darting frequency was higher in trials with a conspecific (mean ± SE = 3.3 ± 0.5) than in individual trials (1.3 ± 0.2). For larvae in the ‘control’ treatment, darting frequency was higher in the presence of a conspecific than in individual trials (Fisher’s LSD, *p* < 0.0001), but for larvae in the ‘risk?’ treatment, darting frequency was similar when tested individually and with a conspecific (*p* = 0.13). Within individual trials, darting frequency did not significantly differ between ‘control’ and ‘risk?’ treatments (*p* = 0.74), whereas within trials with a conspecific, darting frequency was significantly lower in the ‘risk?’ treatment (*p* = 0.04).

## Discussion

Our results demonstrate, in agreement with the previous studies, that *P. cultripes* tadpoles were able to innately recognize predation risk through chemical stimuli from natural predators (larvae of aquatic beetles) feeding on conspecifics, and consequently respond to this threat by displaying antipredator behavior. In addition, we observed that the risk perceived by larvae exposed to the predator stimuli was transferred to unexposed conspecifics in paired trials, which modified their behavior compared to the control group.

In the presence of predator scents, *P. cultripes* tadpoles reduced their activity, spent less time scratching the walls of the experimental bucket, and surfaced to breathe air less often. Amphibian larvae, as many other aquatic organisms, typically reduce activity in response to predators, remaining motionless to avoid being detected and captured (Holomuzki 1995; Kats and Dill 1998; Kiesecker et al. 1996). This antipredator behavior has been observed previously in *P. cultripes* tadpoles in response to other natural predators (Polo-Cavia and Gomez-Mestre 2014; Polo-Cavia et al. 2010). Predation risk also leads to reduced foraging activity in many animals, including anuran larvae (Bridges 2002; Feminella and Hawkins 1994; Jones and Dornhaus 2011; Lima and Dill 1990). Anuran larvae feed mainly on algae, cyanobacteria, and other microorganisms (i.e., periphyton) by scratching the surfaces of submerged rocks and aquatic vegetation (Eklöv and Halvarsson 2000; McDiarmid and Altig 1999; Vitt and Caldwell 2013). Thus, the shorter time devoted by tadpoles to scratching behavior in our experiment might likely reflect a reduction in their foraging activity upon detection of predator cues. The lower surfacing frequency by exposed tadpoles, however, might be a consequence of a lower oxygen demand due to reduced activity, or, alternatively, a direct response to perceived risk of predation preventing encounters with potential predators in the water column. Since frequency of air-breathing covaried with swimming activity (Pearson’s correlation, *r* = 0.47; *F*_1,78_ = 21.84; *p* < 0.0001), the first explanation seems more likely (but see Feder 1983; McIntyre and McCollum 2000).

On the contrary, tadpoles did not alter caudal vibration, position in the water column, or frequency of darting movements in response to predator cues. Rather than serving as a propeller, the terminal filament of the tail of amphibian larvae seems to be primarily used to hold the position in the water column (Du Preez 2015; Minelli and Contrafatto 2009; Touchon and Warkentin 2008). This might explain why caudal vibration was similar between larvae exposed and not exposed to predator chemical cues in our experiment. Also, larvae spent a significant portion of the time near the bottom of the aquaria in individual trials (> 60% overall; Fig. 2D); perhaps, for this reason, we found no differences among predator treatments in the positioning of tadpoles in the water column. As for darting behavior, we expected it to be reduced in the presence of predator cues to avoid conspicuity (Caldwell 1982); however, darting movements were particularly scarce during individual trials, regardless of the risk of predation (Fig. 2F). On the whole, the defensive behavior shown by larvae exposed to predator chemical cues in our first experiment clearly indicates that they perceived the risk of predation, and therefore, could potentially act as risk indicators (i.e., by releasing disturbance cues or by displaying altered behavior) for naïve larvae in trials with a conspecific.

When comparing larval behavior between individual and conspecific trials, larvae paired with a conspecific previously exposed to predator scents tended to reduce their swimming activity, but this was not the case of larvae paired with an unexposed conspecific. The fact that the presence of a conspecific had no effect on tadpoles’ activity per se suggests some degree of risk information transfer. Larvae paired with a conspecific previously exposed to predator cues also reduced caudal vibration, likely as a result of their reduced activity (Pearson’s correlation between swimming activity and tail movement, *r* = 0.80; *F*_1,78_ = 142.34; *p* < 0.0001). Contrary to what was expected, however, scratching behavior of tadpoles was similar in individual and paired trials, regardless of the conspecific’s previous experience with the predator cues. On the other hand, larvae spent more time in the bottom of the experimental aquaria and tended to reduce the frequency of air-breathing in the presence of a conspecific, especially when it was previously exposed to predator cues (Fig. 3D, E). Aquatic prey are known to modify their position in the water column according to perceived risk of predation, increasing substrate use when they detect ambush predators, such as dragonfly nymphs and larvae of diving beetles, which camouflage themselves and attack in the vegetation or near the water surface (Teplitsky et al. 2004; Vitt and Caldwell 2013; Wichard et al. 2002). This finding thus suggests that the presence of startled conspecifics may induce antipredator responses in *P. cultripes* tadpoles. In addition, darting movements of tadpoles increased in the presence of a naïve conspecific (Fig. 3F). If we assume density-dependent effects for tadpoles’ darting behavior (Saxby et al. 2010), then darting frequency of larvae paired with a predator-experienced conspecific was lower than expected. We interpretate this result as the product of larvae increasing darting when sharing the space with a conspecific, but reducing motion in response to social cues potentially informing about risk.

Although some observed effects in our study were just trends, the change in larval behavior in the presence of conspecifics previously exposed to predator cues was consistent and suggests indirect perception of risk by *P. cultripes* tadpoles. It is possible that cues released by threat-exposed larvae (i.e., antipredator behavior and/or disturbance cues) were perceived by naïve conspecifics as a low-risk indicator, in comparison with cues released by predators (kairomones) or injured conspecifics (alarm cues). Previous studies in anurans (Gonzalo et al. 2010; Achtymichuk et al.’s unpublished manuscript) have found weaker behavioral responses to disturbance cues than to alarm cues, which are considered as a more reliable indicator of risk, because they are released only upon tissue damage (Crane et al. 2022). Another factor potentially weakening larval responses in our study is that the startled conspecific was exposed to the disturbance (i.e., the predator cues) previously, but not during the paired trials (since we intended to test for the effect of the entire presence of the conspecific).

While disturbance cues have been proven to induce defensive behavior in naïve conspecifics in several aquatic animals including amphibians (Gonzalo et al. 2010; Griffin 2004; Kiesecker et al. 1999), no studies to date have explored the potential role of disturbance cues in the cultural transmission of acquired information (Crane et al. 2022). Although our experimental design does not allow to discriminate what kind of stimuli (visual and/or chemical) are mediating larval communication, our findings suggest that the risk perceived by *P. cultripes* tadpoles exposed to chemical cues from natural predators can be transferred to naïve conspecifics, which adjust their antipredator behavior in the absence of the threat, presumably using disturbance cues from startled conspecifics as a risk information source and/or by mimicking their peers. This ability of tadpoles to assess predation risk through social cues might play an important role in their interaction with predators, leading to the acquisition or refinement of appropriate defensive responses and increasing survival in predatory encounters.

## Data Availability

The data supporting the findings of this study are available on request from the corresponding author.

## References

[CR1] Bairos-Novak KR, Mitchell MD, Crane AL, Chivers DP, Ferrari MCO (2017). Trust thy neighbour in times of trouble: background risk alters how tadpoles release and respond to disturbance cues. Proc R Soc Lond B.

[CR2] Bairos-Novak KR, Crane AL, Chivers DP, Ferrari MC (2019). Better the devil you know? How familiarity and kinship affect prey responses to disturbance cues. Behav Ecol.

[CR3] Beaupre SJ, Jacobson ER, Lillywhite HB, Zamudio K (2004). Guidelines for use of live amphibians and reptiles in field and laboratory research.

[CR4] Beiswenger RE (1975). Structure and function in aggregations of tadpoles of the American toad, *Bufo americanus*. Herpetologica.

[CR5] Benard MF (2004). Predator-induced phenotypic plasticity in organisms with complex life cycles. Annu Rev Ecol Evol Syst.

[CR6] Blaustein AR, O’Hara RK (1986). Kin recognition in tadpoles. Sci Am.

[CR7] Bridges CM (2002). Tadpoles balance foraging and predator avoidance: effects of predation, pond drying, and hunger. J Herpetol.

[CR8] Brodie ED, Formanowicz DR (1983). Prey size preference of predators: differential vulnerability of larval anurans. Herpetologica.

[CR9] Brown GE, Rive AC, Ferrari MCO, Chivers DP (2006). The dynamic nature of antipredator behavior: prey fish integrate threat-sensitive antipredator responses within background levels of predation risk. Behav Ecol Sociobiol.

[CR10] Burraco P, Díaz-Paniagua C, Gomez-Mestre I (2017). Different effects of accelerated development and enhanced growth on oxidative stress and telomere shortening in amphibian larvae. Sci Rep.

[CR11] Caldwell JP (1982). Disruptive selection: a tail color polymorphism in *Acris tadpoles* in response to differential predation. Can J Zool.

[CR12] Chivers DP, Ferrari MCO (2014). Social learning of predators by tadpoles: does food restriction alter the efficacy of tutors as information sources?. Anim Behav.

[CR13] Chivers DP, Smith RJF (1998). Chemical alarm signaling in aquatic predator-prey systems: a review and prospectus. Ecoscience.

[CR14] Chivers DP, Mirza RS, Bryer PJ, Kiesecker JM (2001). Threat-sensitive predator avoidance by slimy sculpins: understanding the importance of visual versus chemical information. Can J Zool.

[CR15] Clark CW, Harvell CD (1992). Inducible defences and the allocation of resources: a minimalist model. Am Nat.

[CR16] Crane AL, Ferrari MCO, Clark KB (2013). Social learning of predation risk: a review and prospectus. Social learning theory: phylogenetic considerations across animal, plant, and microbial taxa (Animal Science, Issues and Professions).

[CR17] Crane AL, Bairos-Novak KR, Goldman JA, Brown GE (2022). Chemical disturbance cues in aquatic systems: a review and prospectus. Ecol Monogr.

[CR18] Du Preez L (2015). A complete guide to the frogs of southern Africa.

[CR19] Duellman WE, Trueb L (1994). Biology of amphibians.

[CR20] Eklöv P, Halvarsson C (2000). The trade-off between foraging activity and predation risk for *Rana temporaria* in different food environments. Can J Zool.

[CR21] Feder ME (1983). The relation of air breathing and locomotion to predation on tadpoles, *Rana berlandieri*, by turtles. Physiol Zool.

[CR22] Feminella JW, Hawkins CP (1994). Tailed frog tadpoles differentially alter their feeding behavior in response to non-visual cues from four predators. J North Am Benthol Soc.

[CR23] Ferrari MCO, Chivers DP (2008). Cultural learning of predator recognition in mixed species assemblages of frogs: the effect of tutor-to-observer ratio. Anim Behav.

[CR24] Ferrari MCO, Messier F, Chivers DP (2007). First documentation of cultural transmission of predator recognition by larval amphibians. Ethology.

[CR25] Ferrari MCO, Vavrek MA, Elvidge CK, Fridman B, Chivers DP, Brown GE (2008). Sensory complementation and the acquisition of predator recognition by salmonid fishes. Behav Ecol Sociobiol.

[CR26] Ferrari MCO, Wisenden BD, Chivers DP (2010). Chemical ecology of predator-prey interactions in aquatic ecosystems: a review and prospectus. Can J Zool.

[CR27] Goldman JA, Désormeaux IS, Brown GE (2020). Disturbance cues as a source of risk assessment information under natural conditions. Freshw Biol.

[CR28] Gomez-Mestre I, Diaz-Paniagua C (2011). Invasive predatory crayfish do not trigger inducible defences in tadpoles. Proc R Soc Lond B.

[CR29] Gonzalo A, López P, Martín J (2010). Risk level of chemical cues determines retention of recognition of new predators in Iberian green frog tadpoles. Behav Ecol Sociobiol.

[CR30] Gosner KL (1960). A simplified table for staging anuran embryos and larvae with notes on identification. Herpetologica.

[CR31] Griffin AS (2004). Social learning about predators: a review and prospectus. Learn & Behav.

[CR32] Hall D, Suboski MD (1995). Visual and olfactory stimuli in learned release of alarm reactions by zebra danio fish (*Brachydanio rerio*). Neurobiol Learn Mem.

[CR33] Helfman GS (1989). Threat-sensitive predator avoidance in damselfish-trumpetfish interactions. Behav Ecol Sociobiol.

[CR34] Holomuzki JR (1995). Oviposition sites and fish-deterrent mechanisms of two stream anurans. Copeia.

[CR35] Hoppitt W, Laland KN (2013). Social learning: an introduction to mechanisms, methods, and models.

[CR36] Jones EI, Dornhaus A (2011). Predation risk makes bees reject rewarding flowers and reduce foraging activity. Behav Ecol Sociobiol.

[CR37] Kats LB, Dill LM (1998). The scent of death: chemosensory assessment of predation risk by prey animals. Ecoscience.

[CR38] Kiesecker JM, Chivers DP, Blaustein AR (1996). The use of chemical cues in predator recognition by western toad tadpoles. Anim Behav.

[CR39] Kiesecker JM, Chivers DP, Marco A, Quilchano C, Anderson MT, Blaustein AR (1999). Identification of a disturbance signal in larval red-legged frogs, *Rana aurora*. Anim Behav.

[CR40] Lima SL, Dill LM (1990). Behavioral decisions made under the risk of predation: a review and prospectus. Can J Zool.

[CR41] Manassa RP, McCormick MI (2012). Social learning improves survivorship at a life history transition. Oecologia.

[CR42] Manteifel YB, Kiseleva E, Margolis S (2005). An increase in ammonium concentration as a non-specific pheromone signal that is avoided by amphibian larvae. Zool Zhurnal.

[CR43] Martín J, López P (2005). Wall lizards modulate refuge use through continuous assessment of predation risk level. Ethology.

[CR44] McDiarmid RW, Altig R (1999). Tadpoles: the biology of anuran larvae.

[CR45] McIntyre PB, McCollum SA (2000). Responses of bullfrog tadpoles to hypoxia and predators. Oecologia.

[CR46] Minelli A, Contrafatto G (2009). Biological science fundamentals and systematics.

[CR47] Mirza RS, Chivers DP (2002). Behavioural responses to con-specific disturbance chemicals enhance survival of juvenile brook charr, *Salvelinus fontinalis*, during encounters with predators. Behaviour.

[CR48] Peacor SD, Allesina S, Riolo RL, Pascual M (2006). Phenotypic plasticity opposes species invasions by altering fitness surface. PLoS Biol.

[CR49] Peters RA, Clifford CWG, Evans CS (2002). Measuring the structure of dynamic visual signals. Anim Behav.

[CR50] Polo-Cavia N, Gomez-Mestre I (2014). Learned recognition of introduced predators determines survival of tadpole prey. Funct Ecol.

[CR51] Polo-Cavia N, Gonzalo A, López P, Martín J (2010). Predator recognition of native but not invasive turtle predators by naïve anuran tadpoles. Anim Behav.

[CR52] Relyea RA (2004). Fine-tuned phenotypes: tadpole plasticity under 16 combinations of predators and competitors. Ecology.

[CR53] Rivera-Hernández IAE, Crane AL, Pollock MS, Ferrari MCO (2022). Disturbance cues function as a background risk cue but not as an associative learning cue in tadpoles. Anim Cogn.

[CR54] Saxby A, Adams L, Snellgrove D, Wilson RW, Sloman KA (2010). The effect of group size on the behaviour and welfare of four fish species commonly kept in home aquaria. Appl Anim Behav Sci.

[CR55] Schoeppner NM, Relyea RA (2009). Interpreting the smells of predation: how alarm cues and kairomones induce different prey defences. Funct Ecol.

[CR56] Sih A (1997). To hide or not to hide? Refuge use in a fluctuating environment. Trends Ecol Evol.

[CR57] Smith DC, Van Buskirk J (1995). Phenotypic design, plasticity, and ecological performance in two tadpole species. Am Nat.

[CR58] Sokal RR, Rohlf FJ (1995). Biometry.

[CR59] Stauffer H, Semlitsch RD (1993). Effects of visual, chemical and tactile cues of fish on the behavioural responses of tadpoles. Anim Behav.

[CR60] Suboski MD, Bain S, Carty AE, McQuoid LM, Seelen MI, Seifert M (1990). Alarm reaction in acquisition and social transmission of simulated-predator recognition by zebra danio fish (*Brachydanio rerio*). J Comp Psychol.

[CR61] Teplitsky C, Plenet S, Joly P (2004). Hierarchical responses of tadpoles to multiple predators. Ecology.

[CR62] Tollrian R, Harvell CD (1999). The ecology and evolution of inducible defenses.

[CR63] Touchon JC, Warkentin KM (2008). Fish and dragonfly nymph predators induce opposite shifts in color and morphology of tadpoles. Oikos.

[CR64] Van Buskirk J (2009). Natural variation in morphology of larval amphibians: phenotypic plasticity in nature?. Ecol Monogr.

[CR65] Vermeij GJ (1982). Unsuccessful predation and evolution. Am Nat.

[CR66] Vitt LJ, Caldwell JP (2013). Herpetology: an introductory biology of amphibians and reptiles.

[CR67] Wells KD (2010). The ecology and behavior of amphibians.

[CR68] Wichard W, Arens W, Eisenbeis G (2002). Biological atlas of aquatic insects.

[CR69] Wilson DJ, Lefcort H (1993). The effect of predator diet on the alarm response of red- legged frog, *Rana aurora*, tadpoles. Anim Behav.

[CR70] Wilson JC, Detmer TM, White D, Wahl DH (2021). Social influence on anti-predatory behaviors of juvenile bighead carp (*Hypophthalmichthys nobilis*) are influenced by conspecific experience and shoal composition. Hydrobiologia.

[CR71] Wisenden BD (2000). Olfactory assessment of predation risk in the aquatic environment. Philos Trans R Soc B.

[CR72] Wisenden BD, Sorensen P, Wisenden BD (2015). Chemical cues that indicate risk of predation. Fish pheromones and related cues.

[CR73] Wisenden BD, Chivers DP, Smith RJF (1995). Early warning in the predation sequence: a disturbance pheromone in Iowa darters (*Etheostoma exile*). J Chem Ecol.

